# Supplementary material for: Differential chromatin accessibility response to retinoic acid in neuroblastoma with ATRX in-frame-deletions versus ATRX loss-of-function
Source: Neoplasia. 2025 Dec 11;72:101263. doi: 10.1016/j.neo.2025.101263 (PMC12753499; doi:10.1016/j.neo.2025.101263)
Supplement: Supplementary file 1 [file mmc1.docx]

**SUPPLEMENTARY FIGURES**


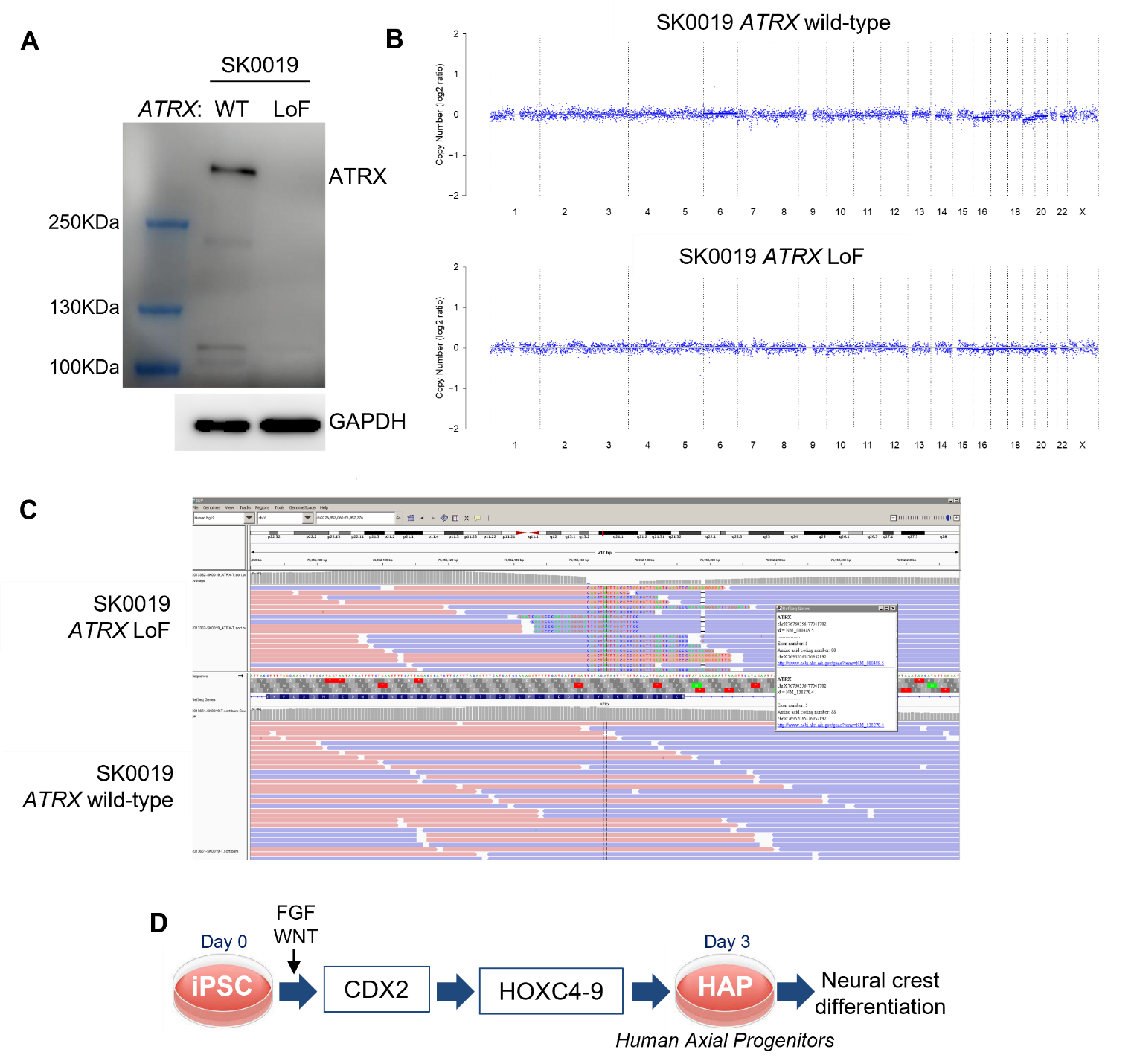


**Supplementary Fig 1:** **(A)** ATRX protein expression in paired SK0019 *ATRX* wild-type and LoF iPSC lines. **(B)** Confirmation of normal karyotype in SK0019 WT and *ATRX* LoF iPSCs by low coverage whole genome sequencing and **(C)** detection of stop codon in exon 5 of *ATRX* by NGS panel sequencing. **(D)** Experimental scheme for differentiation of iPSCs into human axial progenitor cells (HAPs).


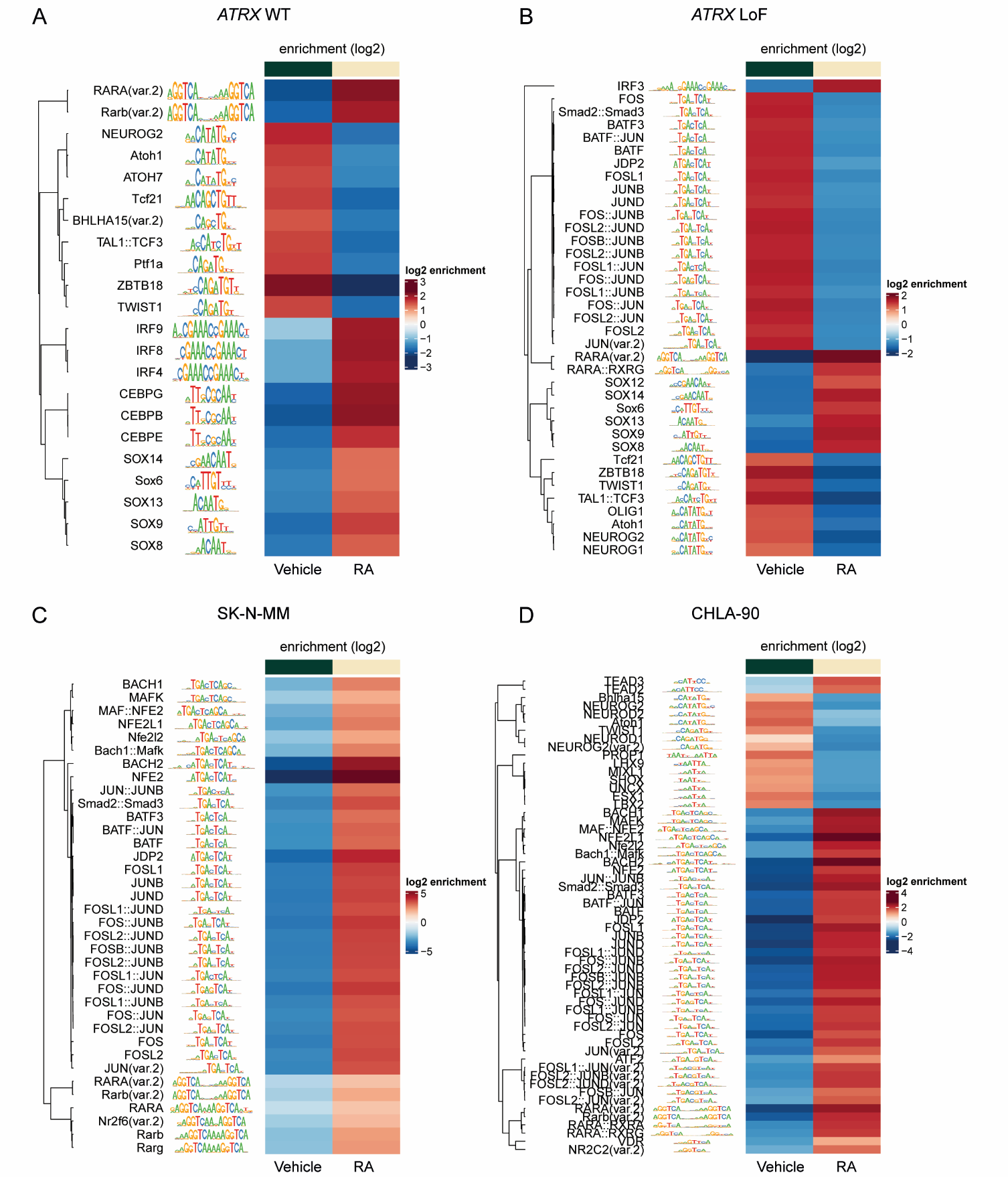


**Supplementary Figure 2:** Heatmaps summarizing most enriched transcription factor binding motifs following treatment with RA of **(A)** p53(2) (*ATRX* WT), **(B)** E6 (*ATRX* KO), **(C)** SK-N-MM (*ATRX* IFF) and **(D)** CHLA-90 (*ATRX* IFF) cell lines.


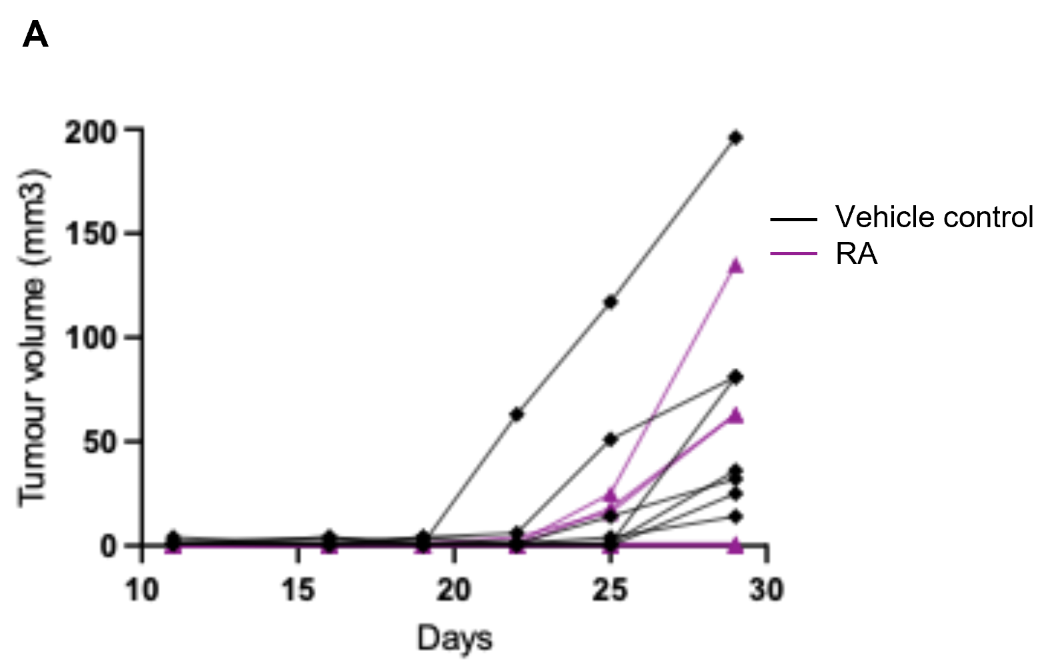


**Supplementary Figure 3: (A)** The IC-pPDX17 PDX (containing an *ATRX* p.Gly1748Arg mutation) was engrafted at day 0 and either RA 53mg/kg or vehicle treatment commenced at D11 post engraftment and tumour volume measured.


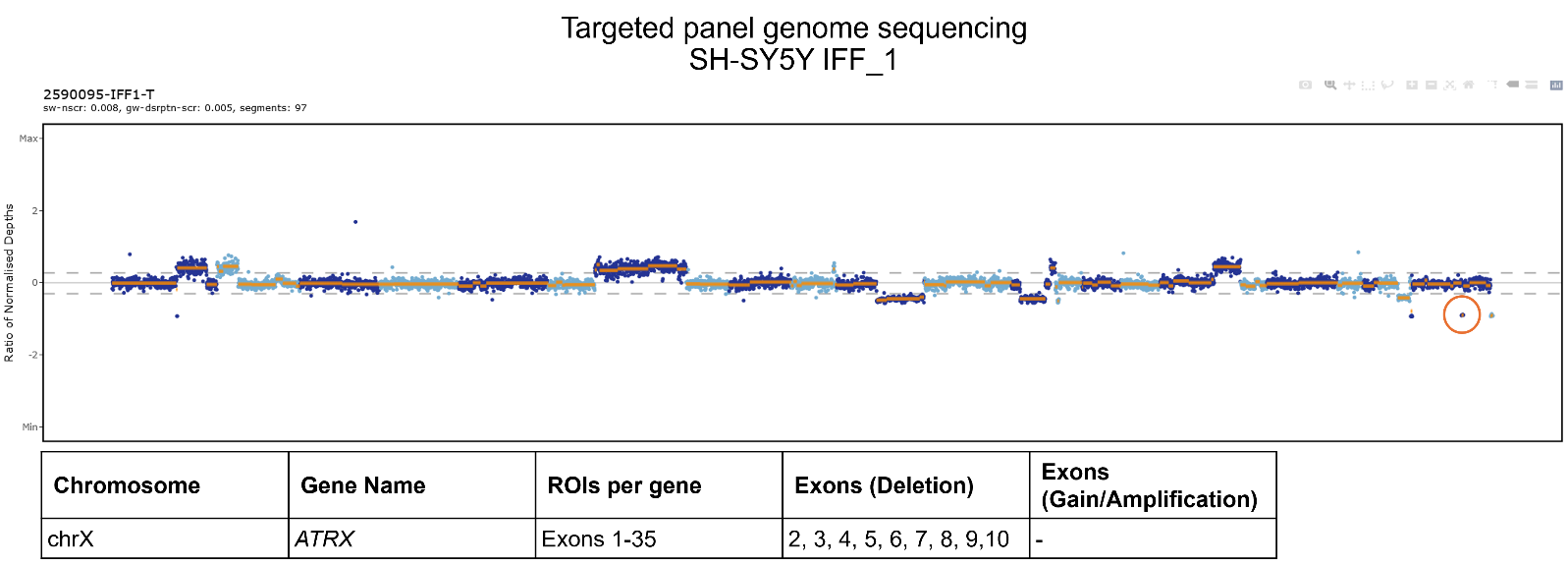


**Supplementary Figure 4:** Targeted-panel genome sequencing of clone SH-SY5Y IFF_1 showing successful deletion of exons 2 to 10 (orange circle) of *ATRX.*
