# Supplementary material for: Differential chromatin accessibility response to retinoic acid in neuroblastoma with ATRX in-frame-deletions versus ATRX loss-of-function
Source: Neoplasia. 2025 Dec 11;72:101263. doi: 10.1016/j.neo.2025.101263 (PMC12753499; doi:10.1016/j.neo.2025.101263)
Supplement: Supplementary file 2 [file mmc2.docx]

**Supplementary methods: List of reagents and resources**

| **Reagent/Resource** | **Reference or Source** | **Identifier or Catalog Number** |
| --- | --- | --- |
| **Experimental Models** |  |  |
| SK0019 iPSC (wild-type and *ATRX* LoF) | Provided by Stephen Scherer, Toronto ^10^ | N/A |
| p53(2) cell line | George et al, EBioMedicine, 2020 ^15^ | N/A |
| E6 cell line | George et al, EBioMedicine, 2020 ^15^ | N/A |
| CHLA-90 | COG cell line repository, USA | RRID:CVCL_6610 |
| SK-N-MM | Provided by Emily Bernstein, USA | RRID:CVCL_C8G1 |
| AMC772 | Provided by Jan Molenaar, Netherlands | N/A |
| IC-pPDX17 | Gudrun Schleiermacher, France ^25^ | N/A |
| SH-SY5Y | ATCC | CRL-2266™ |
| **Antibodies** |  |  |
| ATRX | Sigma | HPA001906  RRID:AB_1078249 |
| GAPDH | Cell signaling | 2118  RRID:AB_561053 |
| H3K27me3 | Cell signaling | C36B11 |
| HRP conjugated anti-rabbit | Dako | PO448 |
| **Oligonucleotides and other sequence-based reagents** |  |  |
| HOXC4 primer | Dickson et al, 2009 ^26^ | N/A |
| HOXC6 primer | Dickson et al, 2009 ^26^ | N/A |
| HOXC9 primer | Dickson et al, 2009 ^26^ | N/A |
| ATAC primers | Corces et al, 2017 ^27^ | N/A |
| **Chemicals, Enzymes and other reagents** |  |  |
| NANOG taqman probe | ThermoFisher Scientific | Hs02387400_g1 |
| OCT4 taqman probe | ThermoFisher Scientific | [Hs04260367_gH](https://www.thermofisher.com/taqman-gene-expression/product/Hs04260367_gH?CID=&ICID=&subtype=) |
| SOX2 taqman probe | ThermoFisher Scientific | Hs04234836_s1 |
| CDX2 taqman probe | ThermoFisher Scientific | Hs01078080_m1 |
| CYP26A taqman probe | ThermoFisher Scientific | Hs00175627_m1 |
| HOXA1 taqman probe | ThermoFisher Scientific | Hs05047045_s1 |
| HOXA4 taqman probe | ThermoFisher Scientific | Hs01573270_m1 |
| GAPDH taqman probe | ThermoFisher Scientific | Hs02786624_g1 |
| PowerUp^TM^ SYBR^TM^Green master mix for qPCR | ThermoFisher Scientific | A25743 |
| Taqman^TM^ Universal PCR master mix | ThermoFisher Scientific | 4304437 |
| Recombinant Human FGF basic (146 aa) protein CF | R&D systems | #233-FB/CF |
| CHIR99021 | Tocris | \|4423 |
| Geltrex basement membrane matrix | ThermoFischer | A1413201 |
| Tagment DNA enzyme and buffer kit | Illumina | 20034197 |
| 13-cis-retinoic acid | Sigma Aldrich | R3255 |
| Tazemetostat | Selleckchem | S7128 |
| MS1943 | MedChemExpress | HY-133129 |
| Maxima First Strand cDNA synthesis kit for RT-qPCR | ThermoFisher Scientific | K1641 |
| ACK lysis buffer | Thermofischer | A1049201 |
| GlutaMAX^TM^ | Thermofischer | 21885025 |
| Ham's F-12 Nutrient Mix | Thermofischer | 15344967 |
| B-27 Supplement^TM^ (50X) minus vitamin A | Thermofischer | 11530538 |
| N-2 supplement | Thermofischer | 17502048 |
| Recombinant Human EGF | PeproTech | AF-100-15 |
| recombinant Human FGF-basic | PeproTech | AF-100-18B |
| recombinant Human IGF-I | PeproTech | 100-11-100UG |
| recombinant Human PDGF-AA and PDGF-BB | PeproTech | #100-13A  #100-14B |
